# 3-Meth­oxy-2-[(*E*)-(4-meth­oxy­phen­yl)imino­meth­yl]phenol

**DOI:** 10.1107/S1600536811000596

**Published:** 2011-01-12

**Authors:** Gonca Özdemir Tari, Şamil Işık, Ramazan Özkan, Ayşen Alaman Ağar

**Affiliations:** a Department of Physics, Faculty of Arts and Sciences, Ondokuz Mayıs University, Kurupelit, TR-55139 Samsun, Turkey; b Department of Chemistry, Faculty of Arts and Sciences, Ondokuz Mayıs University, Kurupelit, TR-55139 Samsun, Turkey

## Abstract

The title compound, C_15_H_15_NO_3_, adopts the enol–imine tautomeric form. The two rings are twisted with respect to each other, making a dihedral angle of 44.08 (5)°. The 3-methoxy-2-[(*E*)-(4-methoxyphenyl)-iminomethyl]phenol unit is almost planar, the largest deviation from the mean plane being 0.047 (2) Å. Such a planar conformation might be related to the occurrence of an intra­molecular O—H⋯N hydrogen bond. In the crystal, inter­molecular C—H⋯O hydrogen bonds link the mol­ecules into sheets parallel to (010). These sheets are inter­connected by weak C—H⋯π inter­actions.

## Related literature

For background to the properties and uses of Schiff bases, see: Barton & Ollis (1979 ▶); Layer (1963 ▶); Ingold (1969 ▶); Cohen *et al.* (1964 ▶); Taggi *et al.* (2002 ▶). For hydrogen-bond motifs, see: Etter *et al.* (1990 ▶); Bernstein *et al.* (1995 ▶). For related structures, see: Özdemir Tarı *et al.* (2010 ▶); Şahin *et al.* (2005 ▶).
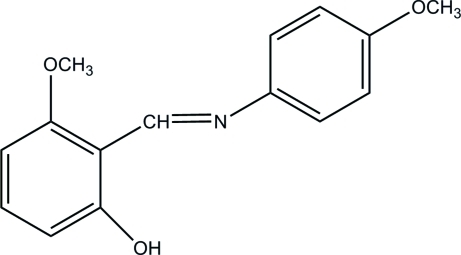


## Experimental

### 

#### Crystal data

C_15_H_15_NO_3_
*M* *_r_* = 257.28
Monoclinic, 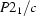

*a* = 14.2658 (8) Å
*b* = 14.1553 (11) Å
*c* = 6.5893 (17) Å
β = 96.398 (9)°
*V* = 1322.3 (4) Å^3^
*Z* = 4
Mo *K*α radiation
μ = 0.09 mm^−1^
*T* = 293 K
0.65 × 0.32 × 0.14 mm

#### Data collection

Stoe IPDS 2 diffractometer
Absorption correction: integration (*X-RED32*; Stoe, 2002) ▶ *T*_min_ = 0.991, *T*_max_ = 0.997
7244 measured reflections
2585 independent reflections
1622 reflections with *I* > 2σ(*I*)
*R*_int_ = 0.032

#### Refinement

*R*[*F* ^2^ > 2σ(*F* ^2^)] = 0.053
*wR*(*F* ^2^) = 0.122
*S* = 1.04
2585 reflections
172 parameters
H-atom parameters constrained
Δρ_max_ = 0.11 e Å^−3^
Δρ_min_ = −0.19 e Å^−3^

### 

Data collection: *X-AREA* (Stoe, 2002) ▶; cell refinement: *X-AREA* ▶; data reduction: *X-RED32* (Stoe, 2002 ▶); program(s) used to solve structure: *SHELXS97* (Sheldrick, 2008 ▶); program(s) used to refine structure: *SHELXL97* (Sheldrick, 2008 ▶); molecular graphics: *ORTEPIII* (Burnett & Johnson, 1996 ▶), *ORTEP-3 for Windows* (Farrugia, 1997 ▶) and *PLATON* (Spek, 2009 ▶); software used to prepare material for publication: *SHELXL97*.

## Supplementary Material

Crystal structure: contains datablocks I, global. DOI: 10.1107/S1600536811000596/dn2646sup1.cif

Structure factors: contains datablocks I. DOI: 10.1107/S1600536811000596/dn2646Isup2.hkl

Additional supplementary materials:  crystallographic information; 3D view; checkCIF report

## Figures and Tables

**Table 1:** Hydrogen-bond geometry (Å, °) *Cg*1 is the centroid of the C1—C6 ring.

*D*—H⋯*A*	*D*—H	H⋯*A*	*D*⋯*A*	*D*—H⋯*A*
C13—H13⋯O2^i^	0.93	2.60	3.428 (3)	149
C14—H14*A*⋯O1^ii^	0.96	2.49	3.412 (3)	162
O2—H2*A*⋯N1	0.82	1.87	2.590 (2)	146
C5—H5⋯*Cg*1^iii^	0.93	2.80	3.486 (2)	132

Symmetry codes: (i) 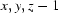; (ii) 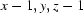; (iii) 
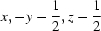
.

**Fig. 1.**
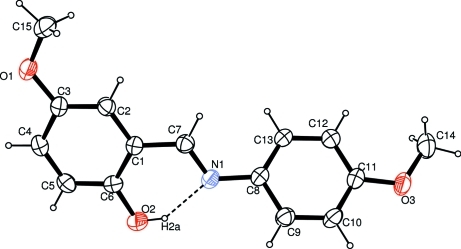
The molecular structure of the title compound, showing the atom-numbering scheme Displacement ellipsoids are drawn at the 30% probability level. H atoms are represented as small sphere of arbitrary radii. Intramolecular H bond is shown as dashed line.

**Fig. 2.**
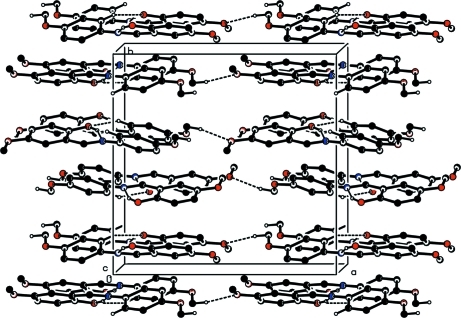
The crystal packing of the title compound showing the formation of sheets parallel to the (0 1 0) plane. H bonds are shown as dashed lines. H atoms not involved in hydrogen bonding have been omitted for clarity.

## supplementary crystallographic information

### Comment

Schiff bases are used as starting materials in the synthesis of important drugs, such as antibiotics and antiallergic, antiphlogistic, and antitumor substances (Barton *et al.*, 1979; Layer, 1963; Ingold 1969). On the industrial scale, they have a wide range of applications, such as dyes and pigments (Taggi *et al.*, 2002). There are two characteristic properties of Schiff bases, *viz*. Photochromism and thermochromism (Cohen *et al.*, 1964). In general, Schiff bases display two possible tautomeric forms, the phenol-imine (OH) and the keto-amine (NH) forms. Depending on the tautomers, two types of intramolecular hydrogen bonds are observed in Schiff bases: O—H···N in phenol-imine (Özdemir Tarı *et al.*, 2010) and N—H···O in keto-amine tautomers (Şahin *et al.*, 2005). Another form of the Schiff base compounds is also known as zwitterion having an ionic intramolecular hydrogen bond (N^+^—H···O^-^) and this form is rarely seen in the solid state (Özdemir Tarı *et al.*, 2010).

The title compound, C_15_H_15_O_3_N_1_, adopts the enol-imine tautomeric form. The C7=N1 [1.278 (3) Å] and C8=N1 [1.419 (2) Å] bond distances are of double-bond character, whereas, C6—O2 [1.356 (2) Å] distance is single bond. These distances are similar to that reported in the literature [1.269 (8) Å] and [1.397 (7) Å] for C=N and [1.332 (8) Å] for C—O respectively (Özdemir Tarı *et al.*, 2010).

The two phenyl rings are twisted with respect to each other making dihedral angle of 44.08 (5)° (Fig. 1). The 4-methoxyphenylimino)phenol moiety is planar with the largest deviation from the mean plane being 0.047 (2)Å at C7. Such planar conformation might be related to the occurrence of the O—H···N intramolecular hydrogen bond (Fig. 1, Table 1). This intramolecular N—H···O hydrogen bond results in the formation of an S(6) ring (Etter *et al.*, 1990; Bernstein *et al.*, 1995).

Intermolecular C—H···O hydrogen bonds link the molecules forming sheets parallel to the (0 1 0) plane (Fig. 2, Table 1). These sheets are interconnected by weak C—H···π interactions (Table 1, *Cg*1 is the centroid of the C1—C6 phenyl ring).

### Experimental

(*E*)-3-methoxy-2-((4-methoxyphenylimino)methyl)phenol was prepared by refluxing a mixture of a solution containing 2-hydroxy-6- methoxybenzaldehyde (15.2 mg, o.1 mmol) in ethanol (30 ml) and a solution containing 4-methoxyaniline (12.3 mg, 0.1 mmol) in ethanol (20 ml). The reaction mixture was stirred for 2 h under reflux. Single crystals of the title compound for *x*-ray analysis were obtained by slow evaporation of an ethanol solution (yield 72%; m.p 346–348 K).

### Crystal data

**Table:**

C_15_H_15_NO_3_	*F*(000) = 544
*M**_r_* = 257.28	*D*_x_ = 1.292 Mg m^−^^3^
Monoclinic, *P*2_1_/*c*	Mo *K*α radiation, λ = 0.71073 Å
Hall symbol: -P 2ybc	Cell parameters from 12079 reflections
*a* = 14.2658 (8) Å	θ = 1.4–27.2°
*b* = 14.1553 (11) Å	µ = 0.09 mm^−^^1^
*c* = 6.5893 (17) Å	*T* = 293 K
β = 96.398 (9)°	Prism, brown
*V* = 1322.3 (4) Å^3^	0.65 × 0.32 × 0.14 mm
*Z* = 4	

### Data collection

**Table:**

Stoe IPDS 2 diffractometer	2585 independent reflections
Radiation source: fine-focus sealed tube	1622 reflections with *I* > 2σ(*I*)
graphite	*R*_int_ = 0.032
Detector resolution: 6.67 pixels mm^-1^	θ_max_ = 26.0°, θ_min_ = 1.4°
φ scan rotation method	*h* = −16→17
Absorption correction: integration (*X-RED32*; Stoe, 2002)	*k* = −17→17
*T*_min_ = 0.991, *T*_max_ = 0.997	*l* = −8→6
7244 measured reflections	

### Refinement

**Table:**

Refinement on *F*^2^	Primary atom site location: structure-invariant direct methods
Least-squares matrix: full	Secondary atom site location: difference Fourier map
*R*[*F*^2^ > 2σ(*F*^2^)] = 0.053	Hydrogen site location: inferred from neighbouring sites
*wR*(*F*^2^) = 0.122	H-atom parameters constrained
*S* = 1.04	*w* = 1/[σ^2^(*F*_o_^2^) + (0.0524*P*)^2^ + 0.1038*P*] where *P* = (*F*_o_^2^ + 2*F*_c_^2^)/3
2585 reflections	(Δ/σ)_max_ < 0.001
172 parameters	Δρ_max_ = 0.11 e Å^−^^3^
0 restraints	Δρ_min_ = −0.19 e Å^−^^3^

### Special details

**Table:**

Geometry. All e.s.d.'s (except the e.s.d. in the dihedral angle between two l.s. planes) are estimated using the full covariance matrix. The cell e.s.d.'s are taken into account individually in the estimation of e.s.d.'s in distances, angles and torsion angles; correlations between e.s.d.'s in cell parameters are only used when they are defined by crystal symmetry. An approximate (isotropic) treatment of cell e.s.d.'s is used for estimating e.s.d.'s involving l.s. planes.
Refinement. Refinement of *F*^2^ against ALL reflections. The weighted *R*-factor *wR* and goodness of fit *S* are based on *F*^2^, conventional *R*-factors *R* are based on *F*, with *F* set to zero for negative *F*^2^. The threshold expression of *F*^2^ > σ(*F*^2^) is used only for calculating *R*-factors(gt) *etc*. and is not relevant to the choice of reflections for refinement. *R*-factors based on *F*^2^ are statistically about twice as large as those based on *F*, and *R*- factors based on ALL data will be even larger.

### Fractional atomic coordinates and isotropic or equivalent isotropic displacement parameters (Å^2^)

**Table:**

	*x*	*y*	*z*	*U*_iso_*/*U*_eq_	
C1	0.20529 (13)	0.11619 (13)	0.4013 (3)	0.0443 (5)	
C2	0.29338 (14)	0.10131 (14)	0.3295 (3)	0.0483 (5)	
H2	0.2959	0.0793	0.1972	0.058*	
C3	0.37566 (14)	0.11898 (15)	0.4526 (3)	0.0516 (5)	
C4	0.37125 (16)	0.15062 (15)	0.6515 (3)	0.0548 (6)	
H4	0.4268	0.1626	0.7353	0.066*	
C5	0.28635 (16)	0.16440 (15)	0.7254 (3)	0.0542 (6)	
H5	0.2848	0.1854	0.8588	0.065*	
C6	0.20294 (15)	0.14735 (14)	0.6034 (3)	0.0479 (5)	
C7	0.11923 (14)	0.10451 (13)	0.2639 (3)	0.0481 (5)	
H7	0.1229	0.0900	0.1274	0.058*	
C8	−0.04619 (13)	0.11307 (14)	0.1930 (3)	0.0459 (5)	
C9	−0.12676 (15)	0.07751 (15)	0.2668 (4)	0.0544 (6)	
H9	−0.1226	0.0522	0.3977	0.065*	
C10	−0.21198 (15)	0.07926 (15)	0.1495 (4)	0.0553 (6)	
H10	−0.2648	0.0535	0.1995	0.066*	
C11	−0.22005 (14)	0.11941 (14)	−0.0446 (4)	0.0512 (5)	
C12	−0.14058 (15)	0.15559 (16)	−0.1200 (4)	0.0561 (6)	
H12	−0.1452	0.1823	−0.2497	0.067*	
C13	−0.05435 (15)	0.15172 (15)	−0.0012 (3)	0.0537 (6)	
H13	−0.0010	0.1755	−0.0526	0.064*	
C14	−0.3216 (2)	0.1629 (2)	−0.3428 (5)	0.0908 (9)	
H14A	−0.3868	0.1590	−0.3970	0.136*	
H14B	−0.2837	0.1297	−0.4310	0.136*	
H14C	−0.3026	0.2280	−0.3338	0.136*	
C15	0.47450 (18)	0.0772 (2)	0.1985 (4)	0.0881 (9)	
H15A	0.5402	0.0742	0.1795	0.132*	
H15B	0.4427	0.1196	0.1000	0.132*	
H15C	0.4472	0.0153	0.1810	0.132*	
N1	0.03849 (12)	0.11390 (12)	0.3283 (3)	0.0496 (4)	
O1	0.46496 (10)	0.10987 (13)	0.3953 (3)	0.0752 (5)	
O2	0.12069 (11)	0.16290 (11)	0.6830 (2)	0.0636 (5)	
H2A	0.0763	0.1500	0.5975	0.095*	
O3	−0.30930 (10)	0.12168 (12)	−0.1453 (3)	0.0662 (5)	

### Atomic displacement parameters (Å^2^)

**Table:**

	*U*^11^	*U*^22^	*U*^33^	*U*^12^	*U*^13^	*U*^23^
C1	0.0417 (11)	0.0426 (10)	0.0480 (12)	−0.0004 (9)	0.0020 (9)	−0.0004 (9)
C2	0.0481 (12)	0.0525 (12)	0.0439 (12)	0.0043 (9)	0.0032 (10)	−0.0031 (9)
C3	0.0418 (11)	0.0593 (12)	0.0524 (13)	0.0040 (10)	0.0000 (10)	0.0011 (10)
C4	0.0537 (14)	0.0603 (13)	0.0475 (13)	−0.0011 (10)	−0.0073 (11)	−0.0002 (10)
C5	0.0630 (15)	0.0575 (13)	0.0412 (12)	0.0010 (10)	0.0017 (11)	−0.0042 (10)
C6	0.0494 (13)	0.0472 (11)	0.0487 (13)	0.0010 (9)	0.0117 (10)	0.0013 (9)
C7	0.0468 (12)	0.0484 (11)	0.0494 (12)	0.0000 (9)	0.0059 (10)	−0.0025 (9)
C8	0.0401 (11)	0.0430 (11)	0.0551 (13)	−0.0016 (9)	0.0075 (10)	−0.0027 (9)
C9	0.0480 (13)	0.0570 (13)	0.0586 (14)	−0.0036 (10)	0.0080 (11)	0.0085 (11)
C10	0.0406 (12)	0.0562 (13)	0.0706 (15)	−0.0078 (10)	0.0127 (11)	0.0063 (11)
C11	0.0404 (11)	0.0493 (11)	0.0632 (14)	−0.0021 (9)	0.0025 (10)	−0.0071 (11)
C12	0.0519 (13)	0.0645 (14)	0.0516 (13)	−0.0045 (10)	0.0046 (11)	0.0036 (10)
C13	0.0402 (12)	0.0667 (13)	0.0548 (14)	−0.0061 (10)	0.0086 (10)	0.0042 (11)
C14	0.0623 (17)	0.125 (3)	0.079 (2)	−0.0102 (16)	−0.0186 (15)	0.0173 (18)
C15	0.0519 (15)	0.142 (3)	0.0722 (18)	0.0053 (15)	0.0146 (14)	−0.0252 (18)
N1	0.0409 (10)	0.0518 (10)	0.0561 (11)	−0.0028 (8)	0.0057 (8)	0.0023 (8)
O1	0.0402 (9)	0.1206 (15)	0.0637 (11)	0.0052 (9)	0.0009 (8)	−0.0167 (10)
O2	0.0557 (9)	0.0812 (11)	0.0560 (10)	0.0037 (8)	0.0157 (8)	−0.0081 (8)
O3	0.0417 (9)	0.0795 (11)	0.0749 (11)	−0.0074 (7)	−0.0055 (8)	0.0016 (9)

### Geometric parameters (Å, °)

**Table:**

C1—C6	1.407 (3)	C9—H9	0.9300
C1—C2	1.407 (3)	C10—C11	1.393 (3)
C1—C7	1.451 (3)	C10—H10	0.9300
C2—C3	1.373 (3)	C11—O3	1.369 (2)
C2—H2	0.9300	C11—C12	1.386 (3)
C3—O1	1.375 (3)	C12—C13	1.384 (3)
C3—C4	1.393 (3)	C12—H12	0.9300
C4—C5	1.369 (3)	C13—H13	0.9300
C4—H4	0.9300	C14—O3	1.419 (3)
C5—C6	1.381 (3)	C14—H14A	0.9600
C5—H5	0.9300	C14—H14B	0.9600
C6—O2	1.356 (2)	C14—H14C	0.9600
C7—N1	1.278 (3)	C15—O1	1.397 (3)
C7—H7	0.9300	C15—H15A	0.9600
C8—C13	1.385 (3)	C15—H15B	0.9600
C8—C9	1.391 (3)	C15—H15C	0.9600
C8—N1	1.419 (2)	O2—H2A	0.8200
C9—C10	1.366 (3)		
			
C6—C1—C2	118.79 (19)	C9—C10—H10	119.9
C6—C1—C7	121.21 (19)	C11—C10—H10	119.9
C2—C1—C7	119.91 (19)	O3—C11—C12	124.9 (2)
C3—C2—C1	120.7 (2)	O3—C11—C10	115.58 (18)
C3—C2—H2	119.6	C12—C11—C10	119.5 (2)
C1—C2—H2	119.6	C13—C12—C11	119.6 (2)
C2—C3—O1	125.3 (2)	C13—C12—H12	120.2
C2—C3—C4	119.3 (2)	C11—C12—H12	120.2
O1—C3—C4	115.40 (19)	C12—C13—C8	121.1 (2)
C5—C4—C3	121.0 (2)	C12—C13—H13	119.5
C5—C4—H4	119.5	C8—C13—H13	119.5
C3—C4—H4	119.5	O3—C14—H14A	109.5
C4—C5—C6	120.4 (2)	O3—C14—H14B	109.5
C4—C5—H5	119.8	H14A—C14—H14B	109.5
C6—C5—H5	119.8	O3—C14—H14C	109.5
O2—C6—C5	118.21 (18)	H14A—C14—H14C	109.5
O2—C6—C1	122.03 (19)	H14B—C14—H14C	109.5
C5—C6—C1	119.75 (19)	O1—C15—H15A	109.5
N1—C7—C1	120.8 (2)	O1—C15—H15B	109.5
N1—C7—H7	119.6	H15A—C15—H15B	109.5
C1—C7—H7	119.6	O1—C15—H15C	109.5
C13—C8—C9	118.6 (2)	H15A—C15—H15C	109.5
C13—C8—N1	123.71 (18)	H15B—C15—H15C	109.5
C9—C8—N1	117.54 (19)	C7—N1—C8	121.78 (19)
C10—C9—C8	120.9 (2)	C3—O1—C15	118.36 (18)
C10—C9—H9	119.6	C6—O2—H2A	109.5
C8—C9—H9	119.6	C11—O3—C14	117.85 (19)
C9—C10—C11	120.29 (19)		
			
C6—C1—C2—C3	−1.5 (3)	N1—C8—C9—C10	176.32 (19)
C7—C1—C2—C3	175.00 (19)	C8—C9—C10—C11	−1.9 (3)
C1—C2—C3—O1	−177.7 (2)	C9—C10—C11—O3	−176.91 (19)
C1—C2—C3—C4	1.0 (3)	C9—C10—C11—C12	1.5 (3)
C2—C3—C4—C5	−0.1 (3)	O3—C11—C12—C13	178.0 (2)
O1—C3—C4—C5	178.7 (2)	C10—C11—C12—C13	−0.3 (3)
C3—C4—C5—C6	−0.2 (3)	C11—C12—C13—C8	−0.6 (3)
C4—C5—C6—O2	−179.4 (2)	C9—C8—C13—C12	0.3 (3)
C4—C5—C6—C1	−0.4 (3)	N1—C8—C13—C12	−174.7 (2)
C2—C1—C6—O2	−179.81 (18)	C1—C7—N1—C8	172.99 (18)
C7—C1—C6—O2	3.7 (3)	C13—C8—N1—C7	−36.1 (3)
C2—C1—C6—C5	1.2 (3)	C9—C8—N1—C7	148.9 (2)
C7—C1—C6—C5	−175.24 (19)	C2—C3—O1—C15	−2.2 (4)
C6—C1—C7—N1	−6.4 (3)	C4—C3—O1—C15	179.1 (2)
C2—C1—C7—N1	177.11 (18)	C12—C11—O3—C14	0.9 (3)
C13—C8—C9—C10	1.0 (3)	C10—C11—O3—C14	179.2 (2)

### Hydrogen-bond geometry (Å, °)

**Table:**

*Cg*1 is the centroid of the C1—C6 ring.

**Table:**

*D*—H···*A*	*D*—H	H···*A*	*D*···*A*	*D*—H···*A*
C13—H13···O2^i^	0.93	2.60	3.428 (3)	149
C14—H14A···O1^ii^	0.96	2.49	3.412 (3)	162
O2—H2A···N1	0.82	1.87	2.590 (2)	146
C5—H5···Cg1^iii^	0.93	2.80	3.486 (2)	132

Symmetry codes: (i) *x*, *y*, *z*−1; (ii) *x*−1, *y*, *z*−1; (iii) *x*, −*y*−1/2, *z*−1/2.
